# Supplementary material for: Complexome profiling of the Chlamydomonas psb28 mutant reveals TEF5 as an early PSII assembly factor
Source: Plant Cell. 2025 Mar 17;37(6):koaf055. doi: 10.1093/plcell/koaf055 (PMC12164590; doi:10.1093/plcell/koaf055)
Supplement: koaf055_Supplementary_Data [file koaf055_supplementary_data.zip › psb28_interactive.zip!/plots/intensity/qualityPlots/peptides/_Cre16.g657350.t1.2.html]

### Cre16.g657350.t1.2

Trivial name:   
SeqCoverage [median]: 37.70  
Mass: 46.38 kDa  
  
 

| Sequence | MissedCleavage | Mass [Da] | wtIntensity | muIntensity | Score |
| --- | --- | --- | --- | --- | --- |
| ALEALLDR | 0 | 899.51 | 1146.52 | 5752.32 | 100.19 |
| ASIETIK | 0 | 760.43 | 0.00 | 3483.77 | 86.87 |
| AVTPSLPSEEVTR | 0 | 1384.72 | 9372.26 | 13857.33 | 104.22 |
| DKETNAEVAIK | 1 | 1216.63 | 394.36 | 646.14 | 63.41 |
| ELQEEQR | 0 | 930.44 | 1304.53 | 1470.96 | 114.19 |
| GAFQSAER | 0 | 864.41 | 511.99 | 576.93 | 87.32 |
| GAFQSAERDK | 1 | 1107.53 | 0.00 | 1513.88 | 50.19 |
| GKGNAGAGMAAVVEED | 1 | 1474.67 | 0.00 | 840.72 | 87.30 |
| IGTPEYMGPELISSR | 0 | 1648.81 | 3590.88 | 9608.57 | 88.44 |
| IQADLGQGHLNIVSADEVILSK | 0 | 2319.24 | 620.09 | 0.00 | 70.99 |
| KPLPEPYESSLKELQEEQR | 2 | 2299.17 | 0.00 | 3563.09 | 41.52 |
| QPATPDFGLQTTHEVIK | 0 | 1880.96 | 0.00 | 5513.23 | 53.78 |
| SIDEQVSK | 0 | 904.45 | 3002.14 | 7190.79 | 109.71 |
| YEVQGQSSKPK | 1 | 1249.63 | 434.34 | 0.00 | 51.84 |
|  |  |  | 20377.1 | 54017.7 |  |
